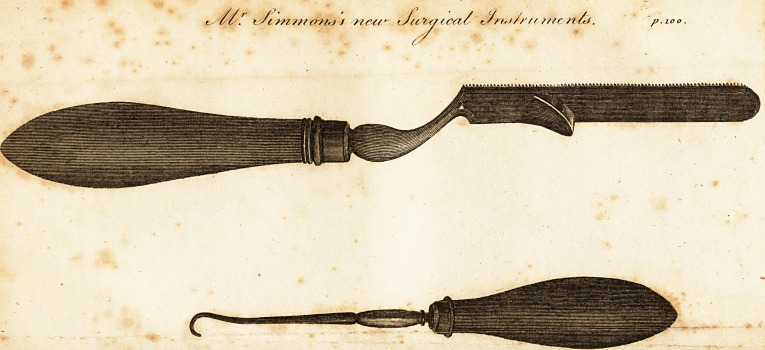# Observations on Select Subjects in Surgery

**Published:** 1805-02-01

**Authors:** 


### Medical and Phyfical Journal.

VOL. XIII.] FEBRUARY 1, 1805. [NO. LXXII.

*Printed for R. PHILLIPS, by W. Thorne, Red Lion Court, Fleet Street, London.*

### OBSERVATIONS ON SELECT SUBJECTS IN SURGERY. BY MR. SIMMONS.

( Continued from our last, pp. 7—10. )

### ON AMPUTATION.

AN historical detail of the material improvements successively introduced into the operation of amputation, would exhibit no bad specimen of the progress of the human mind, in its advancement from rudeness towards refinement. My present object, however, as introductory to the observations that follow, is to offer nothing more than a rapid sketch on the subject.

In the most rude state of the art of surgery, the limb was amputated by a perpendicular incision, without saving either muscle or integument towards covering the wound, and the actual cautery was the means employed to suppress the ensuing hæmorrhage. Such an operation as this, in those ages, would in itself expose the life of the patient to the most imminent hazard, as the hæmorrhage would very commonly be renewed on the exfoliation of the eschar, so as again to require the immediate application of the cautery. A succession of sloughs would in this manner be formed, according to the frequency of its repetition, and the loss of blood on the separation of each, even though not considerable in quantity, more than an enfeebled constitution could well support. With these sources of apprehension, the pain, at times inevitably severe, and long and draining discharge from so extensive an ulcerated surface, would also combine, and render a recovery, under such circumstances, rather an escape than a probable result.

The invention that came first in aid of this class of sufferers, was the securing of the divided arteries with the needle and ligature. For this the world is indebted to Ambrose Pare, who thought himself inspired, so delighted was he when he invented it. Unquestionably, it was a very great improvement; and not unlikely, at a later period, incited Fabricius Hildanus to the use of the cross-stitch; a simple expedient, which, while the muscles shrunk in their dimensions, would gradually approximate the severed integument, and very much accelerate the healing of the wound.

The flap-operation was a still further improvement upon this; but it is to be lamented, that soon after it was first proposed, it fell into disrepute, because several patients on whom it had been performed, died some time after. Yet, as the wound healed in these, surely it would have been more consonant to truth and reason, to ascribe their decease to the ravages of the pre-existing distemper that had led to amputation, than to infer it as the consequence of the new mode of operating.

The prevailing opinions in physiology will ever, more or less, influence the practice of the healing art; and as discoveries are progressively made in those branches of it most nearly allied to medicine, corresponding alterations will be produced in the views and conduct of the practitioner. To the present highly cultivated state of that science we are indebted for union by the first intention, now mostly aimed at after any chirurgical operation, and always I believe after the amputation of a limb. On the previous steps, however, of this operation, surgeons are not so well agreed; therefore, as my opportunities of experience in it have been considerable, I propose to offer a summary of the method which I now pursue. And for the sake of perspicuity, I shall arrange my observations under the following heads. 1. *Division of the soft parts.* 2. *Retraction of the soft parts.* 3. *Division of the bone.* 4. *Securing the arteries.* 5. *Binding up the Limb.* 6. *After-treatment.*

As a preliminary in every amputation, the proper application of the tourniquet need hardly be specified. Many varieties of this instrument are in use among surgeons, but the one which I prefer is delineated and described in the 8th volume of Medical Facts and Observations, edited by Dr. Foart Simmons. It has, at least, equal power, and possesses better security than any other. The tourniquet being properly fixed, we next proceed to the operation itself:

1. Division of the soft Parts. In an amputation at the usual place above the joint of the knee, (from which I shall take my first example) by a single incision I cut at once through the integuments and flexor muscles, in a straight line, line, down to the bone, on the underside of the limb; and then, on the upper side, I complete the annular incision through the integuments *only*. These are next separated by dissection from the extensor muscles, which are now divided in turn down to the bone, likewise by a perpendicular incision, and close to the margin of the everted integument. The mass of muscles underneath is now detached from the bone to a proper distance, to correspond in length to the separated integument above; and lastly, any undivided muscular fibres that may still remain, may be severed by the circular motion of the knife. Of the quantity of muscle and integument together below, and of integument above, proper to be saved, the operator will judge by the rules generally prescribed, as by the union of both it will be his aim to completely cover the end of the stump after the operation. The advantages which I propose to the patient by this simple division or the flexor muscles, are, a quicker execution of this part of the operation; by not separating the integument from the muscles, less, or rather a very moderate degree of suppuration; and, after the healing of the wound, a more even surface, and better bolster to rest upon. Also, I might truly add, that a more speedy cure is thus obtained, than by any other method that I have practised.

But when it is necessary to amputate below the knee, with one exception, considerable variations are introduced. If the limb is moderately full of flesh, and there is reason to believe that the integuments will afford a proper rest in walking, the integuments and muscles should be divided in the customary manner: if, on the contrary, these parts are much extenuated, it will be expedient to form a flap. In forming the flap, my method is to push the point of the amputating knife, in an horizontal direction, at once through the substance of the leg in contact with the tibia and fibula, and then, in a line parallel to these bones, to carry it down to a suitable distance to form a flap of a proper size, when, by a sudden turn of the edge, the knife is made to cut its way almost directly out. On the upper side, I next make a *lunar* section, extending from angle to angle, through the integuments, which are now separated by dissection from the muscles underneath, as in the former instance. The remainder of this part of the operation will thence consist in the division of the extensors or the foot, and any other muscular substance yet undivided.

As by the closure of the extremities of the divided arteries and contraction of a portion of their tubes, the muscles will necessarily shrink to nearly one half of their magnitude at the time of the operation, the flap should be formed sufficiently large at first to allow for this diminution. And the fitness of the *lunar* section of the integument over the tibia, to cover the thick margin of the flap, and save the weight of the body in walking from resting upon new formed skin, is sufficiently obvious. By it the healing of the wound will also be considerably expedited, and its permanency likewise established.

2. Retraction of the soft Parts. Thus far the operation may have succeeded to the wishes of the surgeon; but he will still incur disappointment in the result, unless the retraction of the soft parts be properly executed. For this purpose, different m ethods have been employed: the more simple ones have consisted of a piece of coarse cloth, or pliant leather, with suitable divisions for above or below the knee; and more recently, retractors of tin, or of iron, have been substituted for these. However, all are exposed to the objection, that they hinder a full view of the parts immediately concerned in this stage of the operation; and the latter, in particular, are apt to push the saw below the point marked upon the bone for sawing through it. When this latter accident does occur, the end of the stump is rendered uneven, to the patient's future inconvenience in walking; or, if in a still greater degree, it may terminate in an exfoliation, and prolong the cure. From some trials which I have made with a net-retractor, these several disadvantages may, I think, be obviated. A piece of a common cabbage-net, with suitable divisions in it, will answer the purpose very well. One made of silk may be still better in several respects, though perhaps not very material. But, whether the one or the other be preferred for general use, the meshes should be about a quarter of an inch square.

As the numerous fasciculi of muscular fibres, as well as the muscles themselves, are held together by cellular membrane, pressure upon many distinct points, without making it equably over the whole surface, will effectually keep them back. The operator will thus obtain a distinct view through the interspaces; and he will find a small hook useful to him to expand, narrow, or tighten any particular mesh. *(See the engraving.)*

3. Division of the Bone. Having completed the retraction of the soft parts, we next proceed to saw through the bone. Here especial care should be taken to support the

Dr. Hale's Case of diseased Stomach. Vol. XIII. p. 164.

Printed for Richard Phillips, 6, New Bridge Street.

Mr. Simmons's new Surgical Instruments.

p. 100.

the limb very steadily, and in a direction exactly horizontal; to apply the edge of the saw at a right angle to the bone; and, in working, to bear lightly upon it at the beginning, and again towards the close. These precautions are necessary, to prevent any unevenness at the extremity of the bone, and also to keep it from splintering. And, to avoid any obstruction to the working of the saw, the upper assistant should very gently elevate, while the lower one correspondently depresses that portion of the limb respectively consigned to the care of each. Yet, if after all this assiduity and attention, on the part of the surgeon and his assistants, any splinter or spicula of bone should remain, it may be taken off by the bone-forceps, or blunted by the file. Surgeons are in the habit of using a common file for this purpose; but, on all occasions, and especially in a large muscular limb, a file with a rest for the fore-finger upon the back of it, and a curvature leading to the handle, will be found more convenient. (*See the engraving.*)

4. Securing the Arteries. An important part included in the operation of amputation, is the securing of the divided arteries. These should be very deliberately, and very diligently sought for, drawn out by the tenaculum, and then secured by ligature, firmly but steadily drawn. It was formerly the practice with some surgeons, as they supposed, to prevent it from slipping, and to give additional security, to pass a ligature by means of a needle thro' the extremity of the bleeding vesEel; a precaution that is at least superfluous, as I have never yet been surprized by hæmorrhage, after amputation; and I have never employed any other instrument than the tenaculum.

5. Binding up the Limb.—As we now always aim at union by the first intention, much sedulity is here required. In an amputation above the knee, first removing every thing tight, the integuments should be drawn down, and supported by a few turns of a roller passed round the limb; the blood is then sponged up from the surface of the wound, the muscles are properly disposed, and the ligatures likewise arranged. The lips of the wound are then brought accurately into contact, and in this position secured by strips of adhesive plaster transversely applied. In applying the plasters, care should be taken that they adhere to the lips, which otherwise are apt to retire. Over all, the usual dressings and bandage are now placed, and the patient is carried to bed.

In an amputation below the knee, where the integument

 only only is preserved to cover the end of the stump, the lips of the wound should be retained in contact in a perpendicular direction, or the upper angle of the tibia may force its way through the skin, producing ulceration, and perhaps an exfoliation from the bone. To this direction of the wound, the plasters should be applied also transversely, and the whole secured as in the former instance.

But, when the operator has thought proper to form a flap, the above instructions will be somewhat varied. In adjusting the parts in this case, the portion of integument included in the *lunar* section should be drawn down, so as to form a sort of cup for the reception of the flap, which will then be lifted into its proper receptacle, and retained in it by the adhesive plasters and other dressings.

6. After-Treatment. The operation may now be considered as finished in one of the several ways which I have proposed; yet, to attain success ultimately, much still remains to be done. Alarming accidents may and do supervene after amputation, and are so diversified in their nature, as on some occasions to require an opposite mode of treatment. But, my present observations are meant to apply to that state of the system connected with scrofula. The long continuance of a white swelling prior to amputation, has commonly so impaired the patient's health, and exhausted his strength, as obviously to interdict any means of depletion; and will render it necessary to prevent the loss of blood as much as possible during the operation.

To abate local irritation, and tranquillize the mind, I commonly keep the patient under the gentle influence of opium for a few days, by giving half a grain of the substance, or ten drops of the tincture, every six hours, recollecting to double the evening dose. Where the occasion is less urgent, the full dose at night, and half next morning, will often suffice. The morning dose is chiefly prescribed here to relieve the head-ache, languor, and nausea, left by opium in some constitutions. Were it not sold as a *nostrum*, the *black-drop* might be substituted with advantage for every other internal preparation of opium, as it is free from their unpleasant effects.

In a day or two after the operation, if not contra-indicated by any particular symptom, to support the strength, promote good suppuration, and forward the disposition to heal, the bark may be exhibited. And if it should load the stomach, or act on the bowels, a useful corrective will be found in a few drops of tincture of opium in each dose.

To

To these remedies should also be joined a light and nourishing diet

For the most part, the first dressing should be made on the third day after the operation, and then daily, except the second dressing, which I like to defer another day later, to give time for a more firm union in the parts that may have adhered. But the early dressings should be renewed with great care and exactness; because it is still in the power of the surgeon to correct inadvertency, or supply omission, and to mould the stump into almost any shape. Finally, the ligatures may be expected to come away in ten or twelve days, and the cure completed in two or three weeks; the patient's health and strength recruiting gradually with the healing of the wound.

[To be continued.]

## Figures and Tables

**Figure f1:**